# Stigma: how it affects the substance use disorder patient

**DOI:** 10.1186/s13011-020-00288-0

**Published:** 2020-07-27

**Authors:** Janet Zwick, Hannah Appleseth, Stephan Arndt

**Affiliations:** 1 Editor Substance Abuse Treatment, Prevention and Policy, UCS Healthcare, 4908 Franklin Ave, Des Moines, IA 50310 USA; 2 University of Minnesota Duluth, 320 Bohannon Hall, 1207 Ordean Court, Duluth, MN 55812 USA; 3 Editor Substance Abuse Treatment, Prevention and Policy, Departments of Psychiatry and Biostatistics, JPP, College of Medicine, University of Iowa, Iowa City, IA 52240 USA

## Abstract

The stigma surrounding individuals who have substance use disorders is a pervasive phenomenon that has had detrimental effects on treatment outcomes, health care providers, treatments, research, policies, and society as a whole (Kelly JF, Dow SJ, Westerhoff C , J Drug Issues_40:805-818, Kelly JF, Westerhoff, Int J Drug Policy_21:202-207, 2010). Stigma can be cultivated by various sources, but this article specifically focuses on the impact words have. Individuals influence each other through dynamic language processes. Language, which we use to communicate, represents shared values, history, beliefs, and customs. Moreover, language can be used to promote stigma or decrease it [Snodgrass S: The Power of Words: Changing the Language of Addiction, 2920]. Language usage for addiction medical care is dated in comparison to other standards. Research and organizations are recognizing that substance use treatment, policies, and language need to evolve to aid this crisis and those affected by this disease. Language sustains the stigma surrounding substance use. The stigmatized language used to describe substance use behaviors, individuals with substance use disorders, and substance use treatment can create barriers in essential areas, such as health care, employment, insurance policies, and laws for individuals who are trying to heal and make meaningful contributions to society. There are many ways to contribute to a more accepting society, but it starts with bottom-up processes like language choices in day-to-day conversations. An effort must be made to normalize destigmatized language when referring to substance use and individuals with substance use disorders.

A standard dictionary definition of stigma is a mark of disgrace connected to a situation or quality of a person. A person who experiences stigma is seen as less than others. Stigma is generally based on assumptions or misconceptions. Stigma affects individuals with a substance use disorder, health care providers, treatments, research, policies, and society as a whole.

Language is a combination of words and phrases that set cognitive scripts in motion and create humans’ schemas, which then typically affects humans’ actions [1, 2]. Language describing mental health and addiction has evolved through the years, as we realize the power words carry. Fortunately, we have formally evolved from using terms like “insane asylums”, “lunatics”, “idiots”, or “retards” to describe individuals with mental illnesses. However, there is still progress to be made. Progression is especially necessary in the substance use disorder and addiction realm. The language one uses formulates and contributes to their thoughts, schemas, actions, values, and beliefs; which all combine to create one’s reality. Language influences how society approaches conditions. Currently, language usage for addiction medical care is dated in comparison to other standards.

*Words matter.* Our language helps us understand and interpret the world around us. They convey meaning whether the effect is good or bad. *We can use our words to help decrease stigma* [3]. Person first language recognizes that people are first of all, people. They may have a substance use disorder, so the language becomes person with a subsistence use disorder. Use of the terms abuse, abuser, crazy, addict, dirty or clean describing a toxicology screen or the status of the person, committed suicide, war on drugs, drug habit, “just say no” exemplify a brief preview of how substance use stigma is perpetuated through language.

Kelly et al [2] note that two factors primarily influence stigma. These are cause and controllability. Stigma goes down if people believe that the person did not cause their problem. Historically, the concept of individuals just needing the “willpower to quit using” or “simply pull themselves up by their bootstraps” has been a common perception, which communicates the idea that the individual can control the substance use. Whereas, a destigmatized view would be that this is a disease of the brain, and it is beyond being a matter of self-control and willpower, like how one would think of and treat a physical disease.

Words like “abuse” have associated emotions, and listeners’ reactions have been normed in the general population [4]. Interestingly according to these norms, the word “abuse” has the same emotional valence as the words “asphyxiation” and “HIV.” The term “abuse” is also right between the words homicide and rape. That might make sense when we refer to domestic abuse or child abuse, however, we are talking about a person with a substance use disorder. When we refer to a person as having a substance “abuse” problem, substance abuser, or abusing substances the emotionally laden word carries that word’s emotional valence to the person sending it and the person receiving it.

*There is consistent evidence that using laden words affects how people react to individuals with a substance use disorder. Two studies by Kelly and his colleagues* [2, 5] *suggest that these labels matter. Referring to a person as a “substance abuser”* versus *a person with a “substance use disorder” resulted in subjects offering more punitive judgments and viewing the person’s substance use as willful misconduct. A more recent study* [6] *also suggested that levels of stigma predict more punitive policies and less public health or treatment oriented policies.* “.

In 2017, Michael P. Botticelli, Director of the Office of National Drug Control Policy issued a document titled Changing the Language of Addiction [7]. This document encouraged the Executive Branch agencies to consider the importance in language as related to internal and external communications. The document states that sometimes words used when discussing substance use suggest that the person has a personal failing. He goes on to say the label “person in recovery” has a variety of definitions but usually is in reference to a person who is discontinuing or lessening their use to a healthier level. People in recovery can continue to take medications and receive biopsychosocial services (Botticelli, 2017).

A systematic review [8] based on 28 studies from 2000 to 2011 examined how stigma held by health professionals affects healthcare delivery and found that health professionals typically held negative attitudes towards SUD patients. The healthcare professionals perceived manipulation, violence, and poor motivation as obstructing factors. It was also found that the healthcare professionals had minimal, poor training and education about working with SUD patients. The findings suggested that the healthcare professionals’ negative attitudes caused the patients to have diminished feelings of empowerment, as well as poor healthcare treatment. Health care professionals are often the gatekeepers to treatment for SUD patients, which is why it is imperative that they are adequately trained and educated.

Besides the language of addiction stigma is also associated with individuals in recovery utilizing prescribed medication to assist in the recovery process. Individuals who are taking medications are not “addicted” to it any more than a person with diabetes is addicted to insulin or a person with hypothyroidism is addicted to a thyroid medicine. Individuals who are stabilized on methadone, suboxone or other drugs to treat addiction are not addicted, but are using a medication to help reduce cravings and withdrawal, it restores balance to the brain. It is no different than treating diabetes, high blood pressure or cancer with medication. We do not say individuals with diabetes are addicted to insulin.

With the help of ongoing research, we now recognize that addiction is a chronic brain disorder, not the fault of the addicted individual and that substance use affects the brain in many ways. Gould [9] notes that substances create cognitive issues for people during their withdrawal. Some of the impeded cognitive processes include cognitive flexibility, attention, impulse control, working memory, and learning. Evidence based practices demonstrate that many individuals can be stabilized on medication, accompanied by treatment interventions specifically for addiction. However, a stigma surrounds medication assisted treatment (MAT) due to the beliefs that using medication to treat substance use disorder is just “trading one drug for another”. This stigma could be affecting treatment outcomes, or lack of, for those who could benefit from SUD treatment.

Considering the stigma received, and internalized, by individuals who are suffering in multiple ways and experiencing forms of cognitive impairment it should not come as a surprise that many SUD individuals are in the criminal justice system, are unemployed, have no family or significant other support, have Human Services involvement, are homeless or simply cannot perform the tasks of daily living. Yet these individuals have stigma attached to them due to other problems. Related to cognitive problems, many people who need treatment also need healthcare, housing, food and insurance. Most insurances and other funding do not cover housing, job preparations or food which are basic needs of the person. Researchers at Johns Hopkins Bloomberg School of Public Health showed that the general public did not support employment, housing or insurance policies that benefited people who were dependent on drugs [10].

The Substance Abuse Mental Health Services Administration (SAMHSA) strategic plan for FY 2019-FY 2023 [11] reported that in 2017, 30.5 million Americans reported illicit drug use in the past month and 19.7 million had a substance use disorder in the past year. Additionally, the opioid crisis has affected individuals, families and communities. In 2017, 11.1 million Americans 12 years or older reported misuse of prescription opioids, 900,000 reported heroin use, and 2.1 million had an opioid disorder in the past year. Moreover, 42,000 Americans died from opioid overdoses in 2016.

The World Health Organization noted that in 1999 to 2015 more than 183,000 people in the US died from Opioid overdose and in 2012 more than 250 million opioid prescriptions were written in the US. Canada has also seen an increase in overdose deaths with them increasing fivefold in Ontario between 1991 and 2014 [12]. Stigma continues to be a barrier to individuals seeking help, entering treatment, and accepting medications.

Research and organizations are recognizing that substance use treatment, policies, and language need to evolve to aid this crisis and those affected by this disease. As noted, there are many barriers to treatment, but a major one is access to effective and affordable treatment. Parity legislation and the Affordable Care Act (ACA) in the United States requires insurance companies to cover substance use disorder treatment in the same way they cover similar medical conditions, including medications. *Uncovering the Gaps II* [13] found that over half of the states offered ACA plans in 2017 did not comply with ACA requirements for coverage of substance use disorder benefits. If we are committed to treating addiction as a disease then the stigma related to lack of insurance coverage must be removed (Center for Addiction, 2017).

**Individuals in treatment acknowledge that when family members or friends have suggested they get treatment, the response would be, treatment is for losers and I’m not a loser and don’t need to sit around talking to druggies.**

## What can we do to address stigma?

We can reduce stigma by shifting the view and the visibility of recovering individuals. The community needs to create a visible social identity of recovery and meaningful activity. The David Best study: Jobs, Friends and Houses (JFH) created a visible social identity of recovery and meaningful activity, to assess how stigma is challenged through active and visible community engagement. JFH is a business whose goals are to provide training and employment to those coming out of prison and those with substance problems. All JFH members work on renovating the properties and have attracted outside contracts due to quality and efficiency of work. JFH has a highly visible logo displayed on vans, t-shirts, fleeces and jackets worn by the team. As Wilton and DeVerteuil [14] demonstrated, a highly visible recovery community with celebrations of recovery achievement can change the outlying community’s attitudes and perceptions.

Put a face on the issue of substance use: As noted in the February 10, 2020 edition of the New York Times, Senator Hackman from New York said “stigma is still the largest challenge we face. It prevents people from coming into treatment. The moment was right (for me to speak out). If we’re talking about ending stigma, people like myself have to speak up.”

Other ways to address stigma:

- Educate the public and professionals about substance use disorder and the effects of stigma
- When writing papers and communications be selective about the words you use and be sure to remember people who used substances are, first and foremost, people.
- Speak out about substance use stigma
- Educate the public and professionals about the use of medications for substance use disorder is an evidence based practice when combined with groups and individual sessions.
- Listen, but withhold judgement
- Treat everyone with dignity and respect
- Avoid hurtful or dehumanizing labels
- Demand equality and parity in medical coverage
- Petition government lawmakers for less criminal penalties for people who use drug and other laws that enable stigma
- Use social media to get the message out

As a number of studies have shown, language perpetuates the stigma surrounding substance use. This can create barriers in in vital areas, such as health care, employment, insurance policies, and laws for individuals who are trying to heal and make meaningful contributions to society. There are many ways to contribute to a more accepting society, but it starts with bottom-up processes like language choices. Every person must consciously make an effort to use destigmatized language when referring to substance use and individuals with substance use disorders.
